# Northwestern Pacific Oceanic circulation shaped by ENSO

**DOI:** 10.1038/s41598-024-62361-z

**Published:** 2024-05-22

**Authors:** You-Lin Wang, Fei-Fei Jin, Chau-Ron Wu, Bo Qiu

**Affiliations:** 1 Research Center for Environmental Changes, Academia Sinica, Taipei, Taiwan; 2 Department of Atmospheric Sciences, University of Hawaii at Manoa, Hawaii, USA; 3 Department of Earth Sciences, National Taiwan Normal University, Taipei, Taiwan; 4 Department of Oceanography, University of Hawaii at Manoa, Hawaii, USA

**Keywords:** Physical oceanography, Climate change

## Abstract

The intricate currents of the Northwest Pacific Ocean, with strong manifestations along the westside rim, connect tropical and subtropical gyres and significantly influence East Asian and global climates. The El Niño/Southern Oscillation (ENSO) originates in the tropical Pacific Ocean and disrupts this ocean circulation system. However, the spatiotemporal dependence of the impact of ENSO events has yet to be elucidated because of the complexities of both ENSO events and circulation systems, as well as the increased availability of observational data. We thus combined altimeter and drifter observations to demonstrate the distinct tropical and subtropical influences of the circulation system on ENSO diversity. During El Niño years, the North Equatorial Current, North Equatorial Countercurrent, Mindanao Current, Indonesian Throughflow, and the subtropical Kuroshio Current and its Extension region exhibit strengthening, while the tropical Kuroshio Current weakens. The tropical impact is characterized by sea level changes in the warm pool, whereas the subtropical influence is driven by variations in the wind stress curl. The tropical and subtropical influences are amplified during the Centra Pacific El Niño years compared to the Eastern Pacific El Niño years. As the globe warms, these impacts are anticipated to intensify. Thus, strengthening observation systems and refining climate models are essential for understanding and projecting the enhancing influences of ENSO on the Northwest Pacific Oceanic circulation.

## Introduction

**Figure 1:**
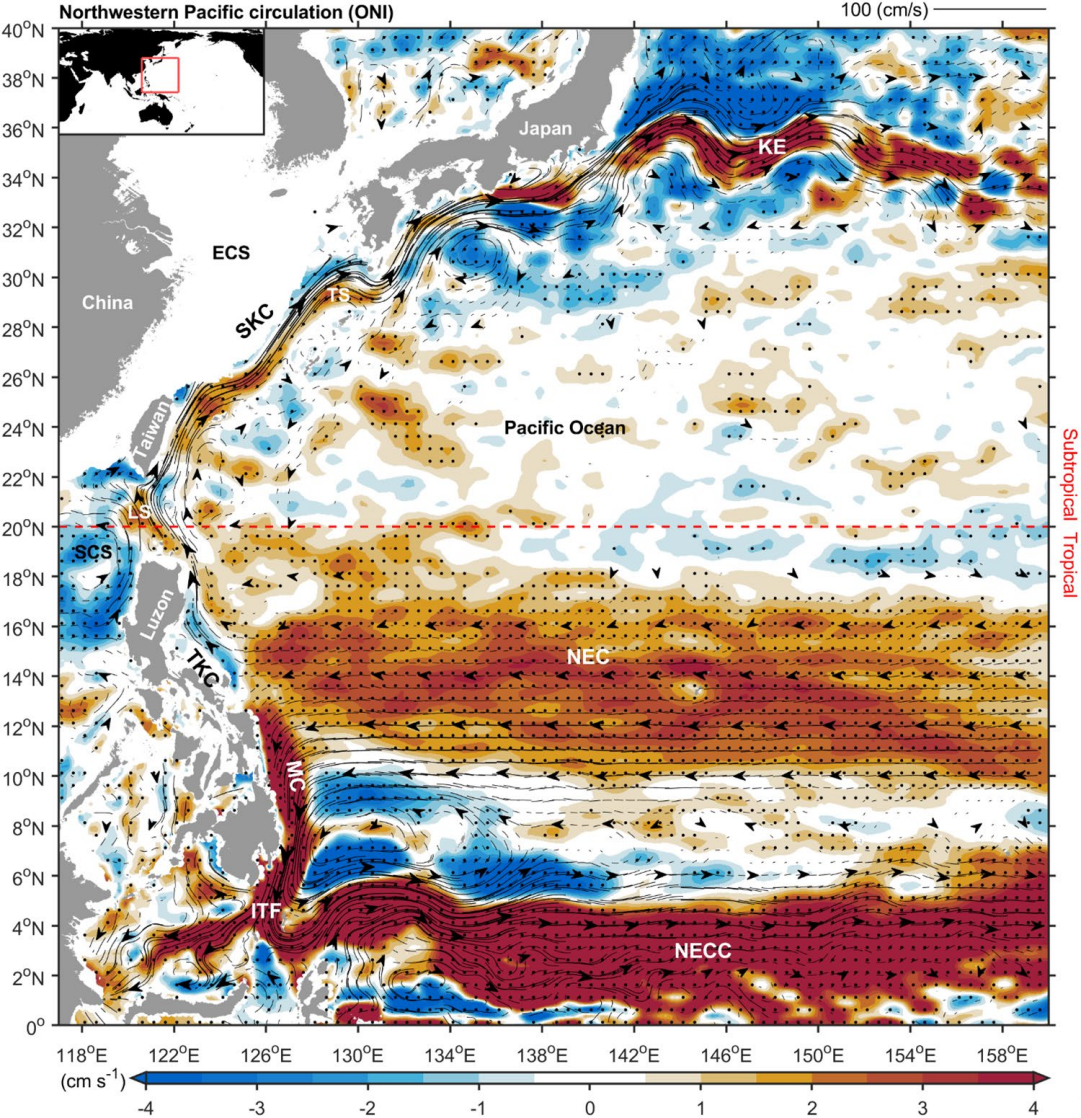
Northwestern Pacific oceanic circulation anomalies are associated with ENSO. Shading shows 1993–2022 surface velocity anomaly regressed onto the Ocean Niño index (ONI). Streamlines and arrows depict the mean sea surface circulation. Black dots signify statistical significance above the 99% confidence level. The red line represents the boundary between tropical and subtropical regions, 20°N. During El Niño, the North Equatorial Current (NEC), Mindanao Current (MC), Indonesian Throughflow (ITF), North Equatorial Countercurrent (NECC), subtropical Kuroshio Current (SKC), and Kuroshio Extension (KE) strengthen. In contrast, the tropical Kuroshio Current (TKC) weakens. The figure was created using MATLAB version R2022b (www.mathworks.com).

The Northwest Pacific Ocean features intricate currents that shape tropical and subtropical gyres (Fig. 1)^1,2^. Within the tropical gyre, the North Equatorial Current bifurcates east of Luzon, forming the tropical Kuroshio Current flowing north and the Mindanao Current flowing south^3,4^. Subsequently, the Mindanao Current merges with the North Equatorial Countercurrent, flowing east into the central Pacific or south via the Indonesian Throughflow into the Indian Ocean^5,6^. In the subtropical gyre, the Kuroshio Current travels north from eastern Taiwan through the East China Sea, reaching the Tokara Strait and forming the Kuroshio Extension in the mid-latitude central Pacific (Fig. 1)^7^. The modern understanding of wind-driven ocean circulation, rooted in Pacific currents, has advanced due to new insights into the impact of the Earth's rotation on gyres and Ekman transport. These insights emphasize the role of wind stress gradients on western boundary currents and define Pacific wind-driven circulations: Tropical and subtropical gyres^2^.

The circulation patterns in the Northwest Pacific Ocean significantly impact East Asia and the global climate^2,8,9^. In the tropics, the Indonesian Throughflow links ocean basins at low latitudes, integrating water from the Mindanao Current via wind-driven circulation (Fig. 1)^6^. It serves as a vital component of thermohaline circulation and is crucial in regulating global climate dynamics^6^. Additionally, the Pacific Ocean hosts the El Niño/Southern Oscillation (ENSO), a key driver of global climate variability. ENSO disrupts weather patterns, affecting ecosystems, agriculture, cyclones, droughts, wildfires, and floods worldwide^10–12^. The Pacific tropical gyre transports masses toward the equator, greatly influencing the Western Pacific warm pool, ENSO life cycle, and Asian monsoon system^2,13^. In the subtropics, the interaction between mid-latitude cold air and warm water along the Kuroshio Extension facilitates the exchange of heat and moisture between the ocean and the atmosphere, thus influencing winter storm tracks^14^. Moreover, North Pacific coupled ocean–atmosphere dynamics regulate El Niño development: The atmospheric response to the Kuroshio Extension triggers the Pacific Meridional Modes that drive the ENSO, and ENSO teleconnections reinforce the extratropical atmospheric forcing of the Kuroshio Extension^15^. These findings have highlighted the importance of the Northwest Pacific Ocean circulation. Consequently, exploring the response of the Northwest Pacific Ocean circulation under the influence of climate change has attracted increased attention.

On the interannual timescale, extensive research findings have suggested that ENSO exerts a dominant influence on regulating Northwest Pacific Ocean circulation^2,16^. In the tropics, El Niño strengthens the North Equatorial Countercurrent^17–20^ and North Equatorial Current^21–23^. Conversely, the tropical Kuroshio Current east of Luzon strengthens during La Niña years and weakens during El Niño years^2,24^. Shifting the bifurcating latitude of the North Equatorial Current (NECBL) northward directs more seawater southward, strengthening the Mindanao Current, while reduced northward flow weakens the Kuroshio Current^2,4,22,25,26^. El Niño-induced wind changes generate coastal Kelvin waves through the Indonesian Throughflow into the Indian Ocean^27^. The El Niño-related Walker Circulation shifts longitudinally, impacting the variability in Indonesian throughflow transport^28^. In the subtropics, the Kuroshio Current transport southeast of Taiwan weakened during the 1997–1998 El Niño^29^. However, conflicting results from other observations persist. According to the Acoustic Doppler Current Profiler (ADCP) moorings, the North Equatorial Countercurrent significantly weakened during 2014^30^, coinciding with a weak El Niño event^31^ or as a precursor to the stronger El Niño years of 2015–2016^32^. A 20-year altimeter dataset indicated no notable relationship between the Kuroshio Current near 18°N and El Niño^24^. Furthermore, the Kuroshio Current northeast of Taiwan is strengthened during El Niño years^33^. These contradictory observational results regarding how El Niño events impact Northwest Pacific Ocean circulation require further clarification. The ENSO is characterized by its inherent complexity and diversity^34^. Moreover, the circulatory system in the Northwest Pacific exhibits significant variability (Extended Data Fig. 1). Elucidating the interconnections between El Niño events and the Northwest Pacific circulation dynamics requires detailed investigation.

## Changes in Northwestern Pacific Ocean circulation

In the tropical Pacific, the circulation system is highly vulnerable to El Niño impacts, given that this region is the epicenter of ENSO. To demonstrate the influence of El Niño on the surface circulation of the tropical Pacific Ocean, we calculated surface velocity anomalies by removing trends and the annual cycle. These anomalies were regressed onto the Niño 3.4 index and are shown in Fig. 1 and Extended Data Fig. 2.

**Figure 2:**
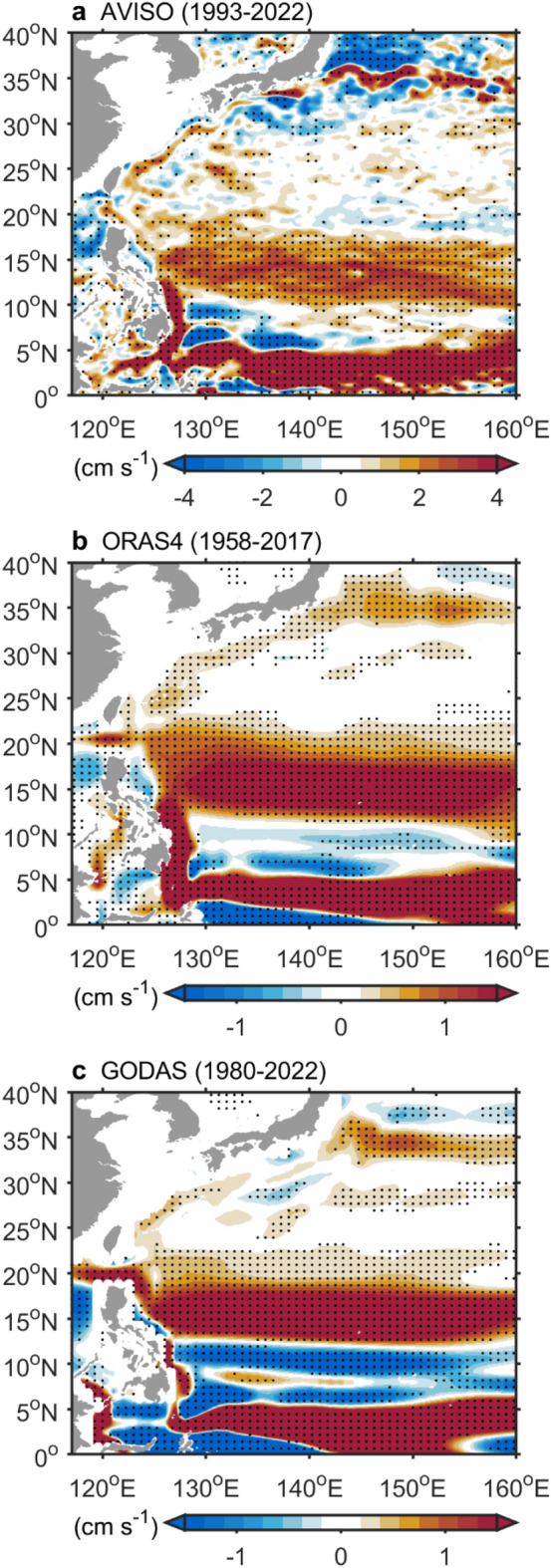
Shading shows surface velocity anomaly from AVISO (**a**), ORAS4 (**b**), and GODAS (**c**) regressed onto the ONI. The upper 200 m depth velocity data from ORAS4 and GODAS were used. Black dots indicate statistical significance above the 99% confidence level. The figure was created using MATLAB version R2022b (www.mathworks.com).

Between 10°N and 18°N, from east of Luzon Island to longitude 160°E, the North Equatorial Current exhibited a 4 cm/s increase in westward velocity during a typical El Niño (when the Niño 3.4 index warms by 1 °C) (Fig. 1), representing 11–20% of the climatological speed in the North Equatorial Current (20–35 cm/s, Extended Data Fig. 2). Conversely, during La Niña years, the pattern reversed. Downstream of the North Equatorial Current, the tropical Kuroshio Current (14.5°N–16°N) weakened by 5–10% (reduced to − 2.5 cm/s compared to its average speed of 20–30 cm/s), while the Mindanao Current strengthened by 20–25% (increased to + 20 cm/s compared to its average speed of 75–85 cm/s) (Fig. 1 and Extended Data Fig. 2). Previous studies have linked these changes to the NECBL: When the NECBL shifts farther north during El Niño, more seawater flows south into the Mindanao Current, enhancing it, and less seawater flows north into the Kuroshio Current, weakening it^3,25^.

Additionally, during El Niño events, the Indonesian Throughflow increased by 10–15% (123°E, 3.2°N, + 5 cm/s), while the North Equatorial Countercurrent surged by 25–30% (east of 135°E, + 15 cm/s) (Fig. 1 and Extended Data Fig. 2). We confirmed that El Niño events intensified the North Equatorial Current, Mindanao Current, North Equatorial Countercurrent, and Indonesian Throughflow, as well as weakened the tropical Kuroshio Current (Fig. 1 and Extended Data Fig. 2), and vice versa during La Niña years. These responses in tropical ocean currents aligned with previous observational findings^2,16^.

However, in the subtropical region, our findings differed from those of previous studies. In Fig. 1 and Extended Data Fig. 3, we observed 3–4% surface strengthening in the Kuroshio Current from east of Taiwan to the East China Sea and the Tokara Strait (+ 3 cm/s, compared to the average speed of 80–90 cm/s). Additionally, from southeast Japan to the meandering region, the Kuroshio Current intensified by approximately 9–12% (135°E to 150°E, + 6 to + 12 cm/s, compared to the average speed of 70–100 cm/s) (Extended Data Fig. 4). In general, the NECBL is considered a convenient indicator for representing the Kuroshio Current intensity. When the NECBL shifts northward during El Niño, the reduced inflow of seawater weakens the Kuroshio Current^2,16^. However, we found that both the subtropical Kuroshio Current and its meandering region were strengthened during El Niño events. These contradictory findings might be due to early observational data being predominantly focused on the tropical Kuroshio Current^2,24,35^ and being limited spatiotemporally, making it challenging to observe that the intensity of the subtropical Kuroshio Current is not negatively correlated with El Niño.

**Figure 3:**
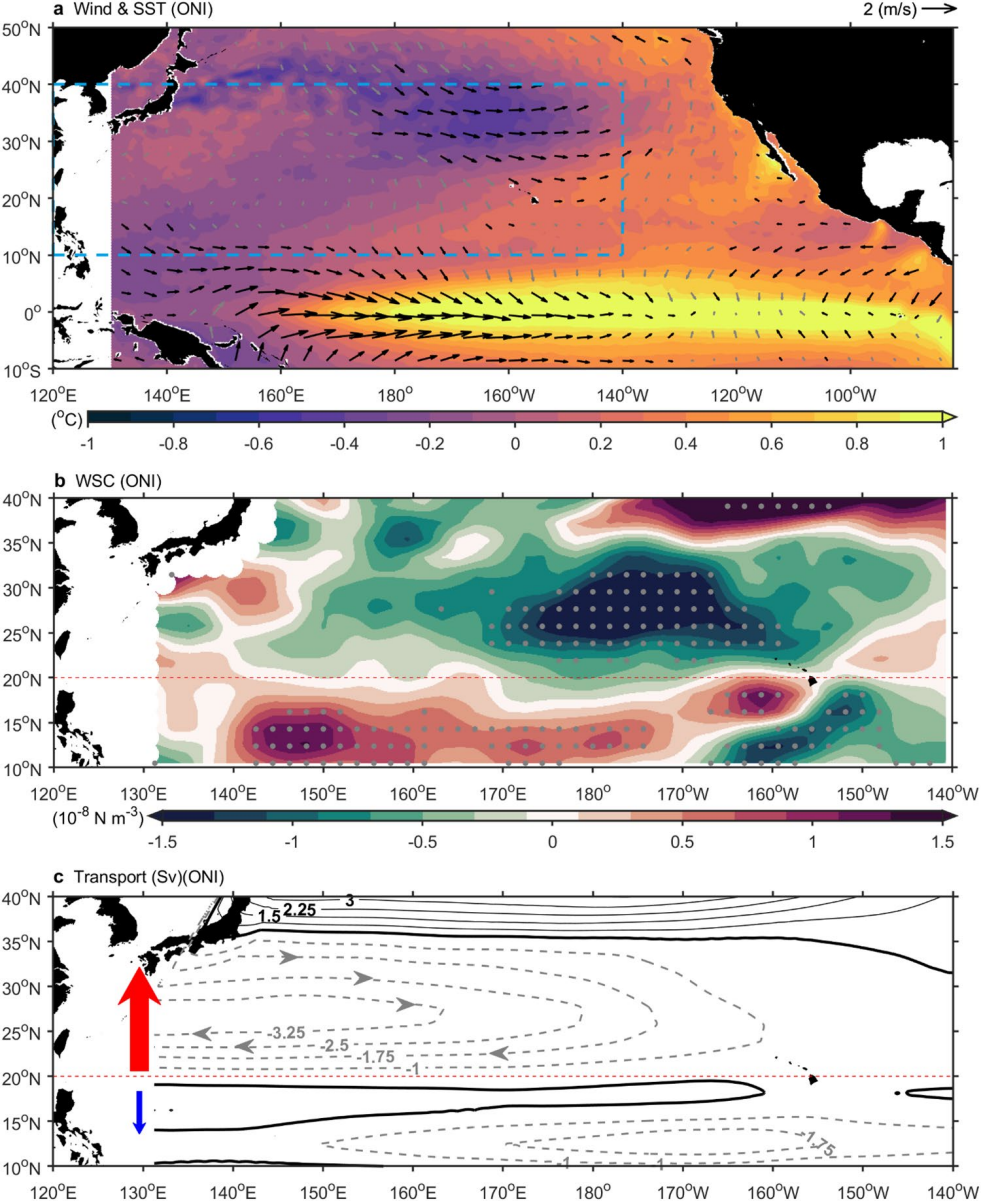
El Niño impacts North Pacific subtropical ocean circulation through changes in basin winds. (**a**), Shading shows 1993–2022 sea surface temperature anomaly regressed onto ONI. Black and gray arrows indicate statistically significant 10 m wind regressions above and below the 99% confidence level. The blue box represents the plotting range of Figs. b and c (120°E–140°W, 10°N–40°N). (**b**), Shading represents 1993–2022 wind stress curl anomaly linear regression onto ONI, with gray dots indicating statistical significance above the 99% confidence level. (**c**), Contour lines represent linear regression of 1993–2022 North Pacific Sverdrup transport onto the ONI index (contour interval: 0.75 Sv). Negative values indicate southward transport, while positive values represent northward transport. Red arrows indicate enhanced subtropical Kuroshio, and blue arrows indicate weakened tropical Kuroshio. (**b**,**c**) The red dashed line in both figures marks the boundary between tropical and subtropical regions at 20°N. The figure was created using MATLAB version R2022b (www.mathworks.com).

**Figure 4:**
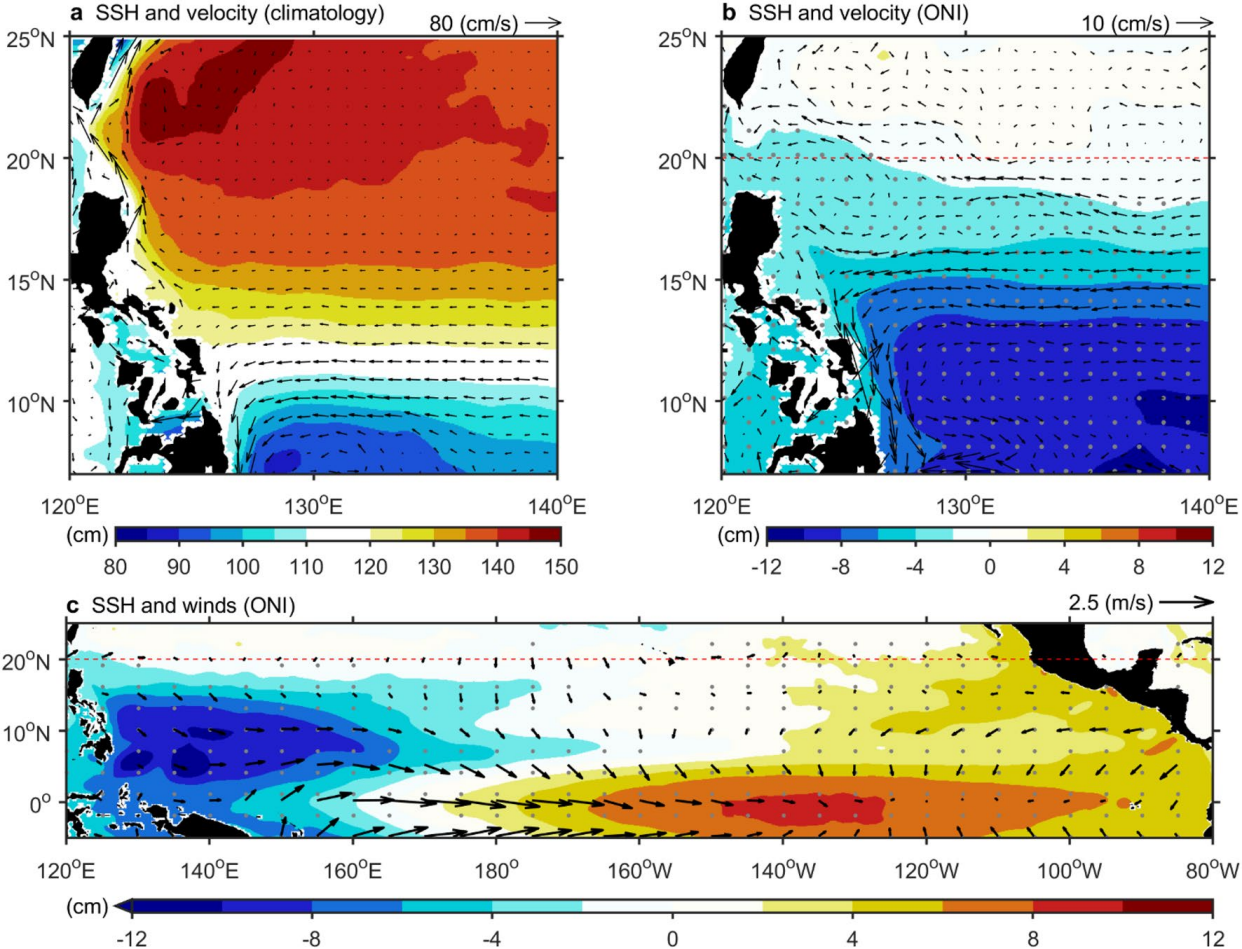
El Niño affects the Northwest Pacific tropical circulation through changes in Pacific Warm Pool sea surface height (SSH) associated with trade winds. (**a**), Shading represents 1993–2022 SSH anomaly regressed onto ONI. Vectors indicate mean surface velocity. (**b**,**c**) Shading shows SSH anomaly regressed onto ONI in TKC, NEC, and MC regions (**b**), and in tropical Pacific region (**c**). Dots indicate statistical significance above the 99% confidence level. (**c**) Vectors show 1993–2022 10 m winds anomaly regressed onto ONI. The red dashed line in both figures marks the boundary between tropical and subtropical regions at 20°N. The figure was created using MATLAB version R2022b (www.mathworks.com).

To supplement the lack of observational data on the subtropical Kuroshio Current, we analyzed surface drifter data from 1979 to 2022, covering both the subtropical Kuroshio Current and its meandering region spatially (Extended Data Figs. 5 and 6). During El Niño, surface drifter data revealed a 5–10 cm/s strengthening of the subtropical Kuroshio Current (Extended Data Fig. 5). Moreover, the Kuroshio Extension region strengthened by 18 cm/s (Extended Data Fig. 6). These results corroborated the overall enhancement of the Kuroshio Current, with weakening observed only in its upstream tropical region during El Niño (Fig. 1). Notably, the surface drifter observations indicated stronger Kuroshio Current variations during El Niño events than the satellite data did (Extended Data Figs. 5 and 6). The speeds of the drifters were 2–3 times faster than those of the AVISO data because of the following factors: (1) AVISO data have coarser temporal and spatial resolutions than the drifter data. (2) Drifters measure surface currents at 15 m depth, whereas AVISO data represent the geostrophic component of the upper ocean. Due to the shorter duration of AVISO data, there may be a limited number of El Niño events. Therefore, we analyzed two long-term datasets, ORAS4 (1958–2017) and GODAS (1980–2022). The results (Fig. 2) from both ORAS4 and GODAS supported the conclusion that during El Niño years, the subtropical Kuroshio strengthens while the tropical Kuroshio weakens.

**Figure 5:**
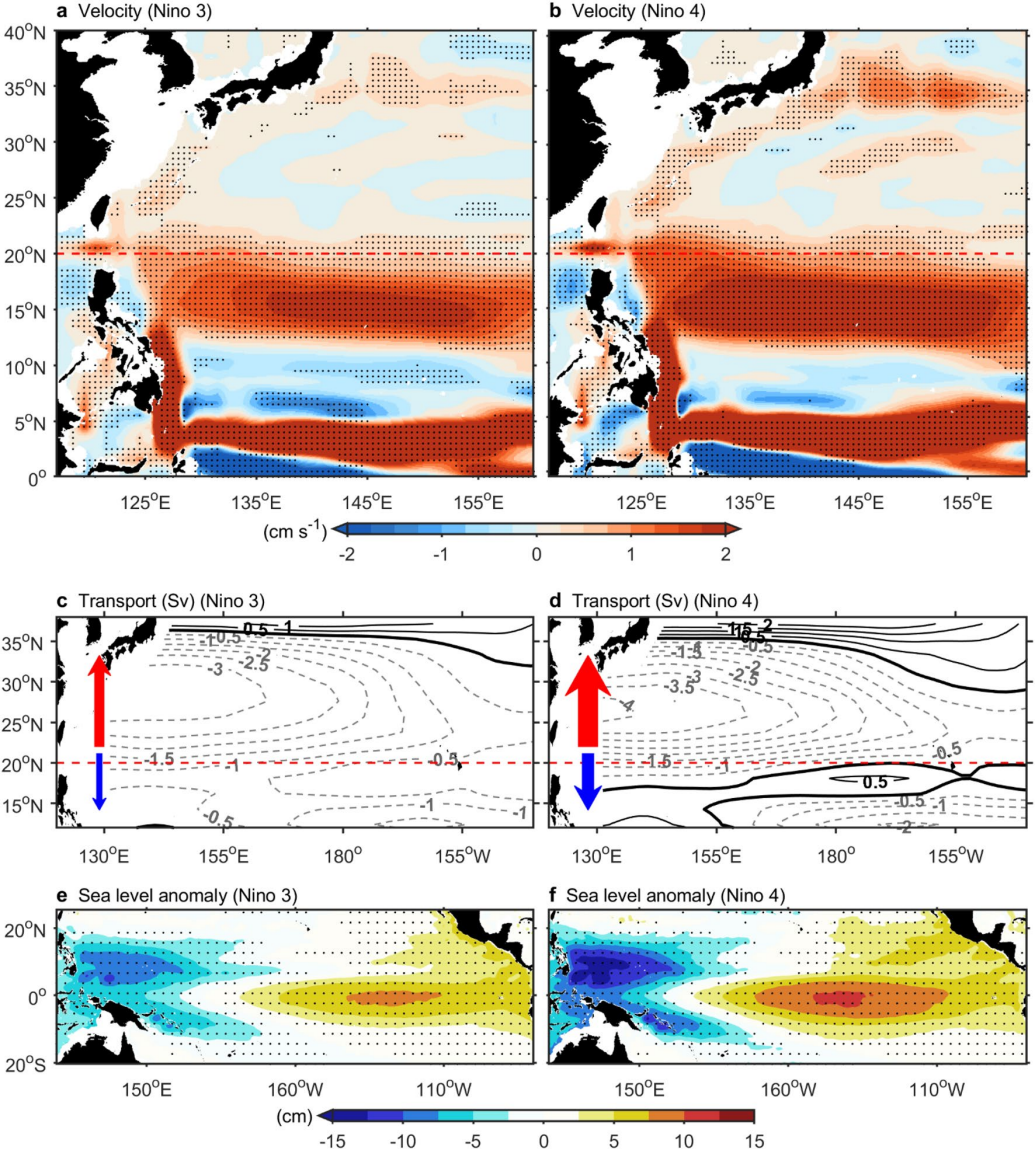
(**a**) Shading shows the sea surface velocity anomaly (ORAS4, 1958–2017, upper 200 m) regressed onto Niño 3 (a proxy for the EP El Niño). (**b**) Similar to (**a**) but using the Niño 4 index (a proxy for CP El Niño events). (**c**) Contours showing the regression of the North Pacific Sverdrup transport (NCEPr1, 1950–2022) onto the Niño 3 index (interval: 0.5 Sv). Negative values: Southward transport; positive values: Northward transport. (**d**) Similar to (**c**) but using the Niño 4 index. (**e**) Shading shows the SSH anomaly (AVISO, 1993–2022) regressed onto the Niño 3 index. (**f**) Similar to e but using the Niño 4 index. (**a**–**d**) The red dashed line marks the boundary between tropical and subtropical regions at 20°N. (**a**,**b**) and (**e**,**f**) Black dots indicate statistical significance above the 99% confidence level. The figure was created using MATLAB version R2022b (www.mathworks.com).

**Figure 6:**
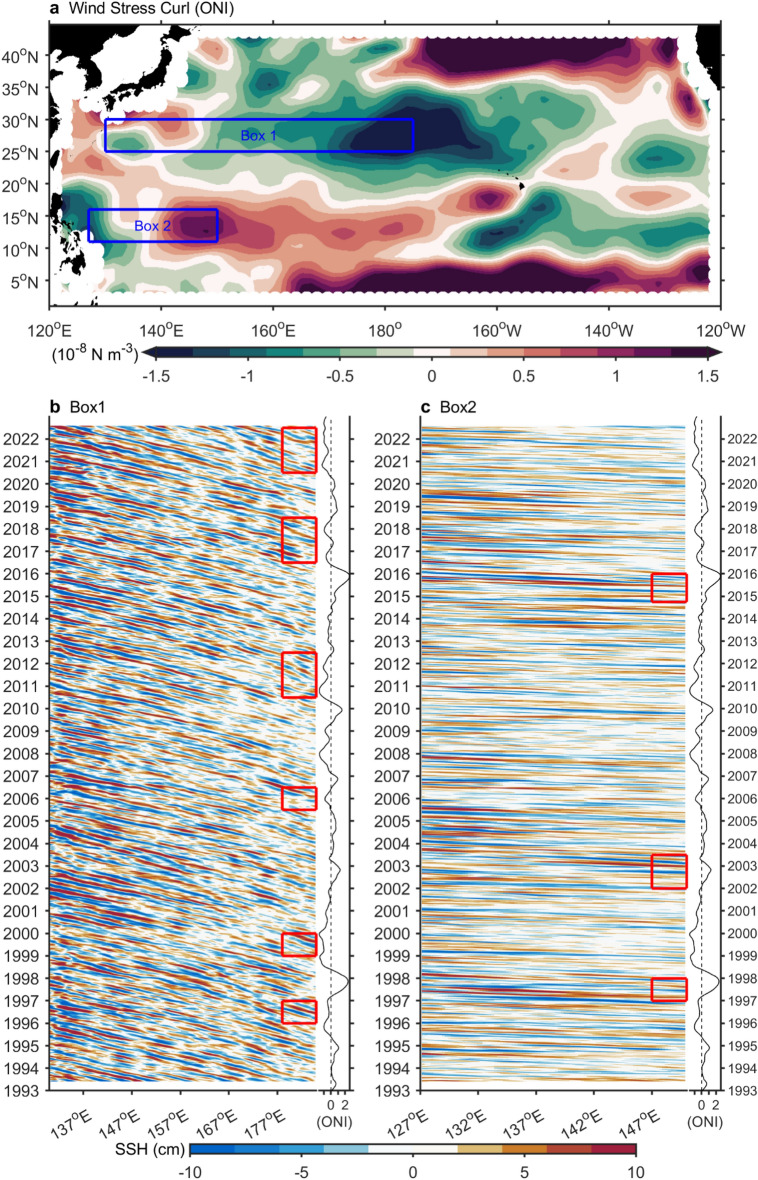
Basin winds and Rossby wave. (**a**) 1950–2022 wind stress curl anomaly regressed onto the ONI index. Box1 (130E to 185E, 25^o^N-30^o^N) and Box2 (127E to 150E, 11^o^N-16^o^N) indicate regions where Rossby waves induced by ENSO associated wind stress curl anomaly. (**b**) Hovmöller diagram of sea surface height anomaly (SSHA) in Box1. (**c**), Same as (**b**), but in Box2. A 300-day high-pass filter has been applied^51^. The figure was created using MATLAB version R2022b (www.mathworks.com).

In general, our findings indicated that the NECBL can represent only the tropical Kuroshio Current. A new explanation is needed to understand how El Niño events affect the subtropical Kuroshio Current.

## Subtropical versus tropical dynamics

In the context of wind-driven ocean circulation theory, the shifts observed in the two major wind systems of the North Pacific, the trade winds and the mid-latitude westerlies, during El Niño years were potential drivers that impacted the subtropical Kuroshio Current. The trade winds accumulate warm water in the western Pacific warm pool and cause upwelling of colder water in the east, strengthening the air pressure difference across the basin and driving trade winds via Bjerknes feedback^36,37^. During El Niño, equatorial trade winds weaken, increasing the western Pacific pressure and lowering the eastern Pacific pressure. Warm water moves east, reducing upwelling and leading to central and eastern Pacific warming (shading in Fig. 3a). Weakened trade winds and warming SSTs reinforce each other, amplifying El Niño development via Bjerknes feedback. In the subtropics, El Niño induces teleconnections with the extratropics by triggering sea level pressure (SLP) variations, and these variations are marked by a deepened Aleutian low through large-scale atmospheric Rossby waves^38,39^. The deepening Aleutian low strengthens midlatitude westerlies (30°N–40°N, vector in Fig. 3a), causing sea surface temperatures to cool during El Niño (purple in Fig. 3a).

The weakened trade winds and strengthened westerlies during El Niño alter the wind stress curl in the Pacific subtropical region, which is known to shape the intensity of the subtropical Kuroshio Current^40^. Climatologically, a negative wind stress curl occurs between tropical trade winds and mid-latitude westerlies, driving southward flow beneath and compensating for the northward-flowing Kuroshio Current. During El Niño, weakened tropical trade winds generated positive wind stress curl anomalies on their northern flank (10–18°N) (Fig. 3b). Conversely, strengthened mid-latitude westerlies created negative wind stress curl anomalies on their southern flank (22°N–35°N) (Fig. 3b). We computed the Sverdrup transport by zonally integrating the north‒south water transport caused by the wind stress curl (Fig. 3c). In El Niño years, the negative anomaly of the subtropical wind stress curl generated by strengthened mid-latitude westerlies contributed to an approximately 11% enhancement (+ 3.25 Sv, Fig. 3c) of the subtropical Kuroshio Current transport (compared to the average transport of approximately 30 Sv^41^). The surface velocity observed by the surface drifters also increased by approximately 10% (+ 9 cm/s, Extended Data Fig. 5). These results indicated that the El Niño-related strengthening of westerlies results in a negative anomaly in the subtropical wind stress curl, further enhancing the subtropical Kuroshio Current.

In the tropics, the influence of the wind stress curl on the tropical Kuroshio Current led to a modest reduction of 0.5 Sv during El Niño events (Fig. 3c), implying that this factor alone was not the primary cause for the weakening of the tropical Kuroshio Current. Typically, the strength of the Kuroshio Current was maintained through geostrophic balance, where higher sea levels in the interior Pacific and lower sea levels in marginal seas had a function (Fig. 4a). During El Niño, the sea level decrease in the warm pool reduced the sea surface height difference across the Kuroshio Current, weakening it (Fig. 4b). Conversely, the decrease in sea level south of NEC increased the sea surface height across NEC, strengthening it (Fig. 4b). The sea level drop on the eastern side of the Mindanao Current enhanced its southward geostrophic component, reinforcing the Mindanao Current (Fig. 4b).

These changes in tropical ocean currents were closely linked to sea level variations in the North Pacific warm pool (Fig. 4c). Under normal conditions, equatorial trade winds pushed warm water westward, forming a warm pool. During El Niño, weakened trade winds caused the warm pool water to move eastward, resulting in abnormal sea level drops (Fig. 4c). In addition to the NECBL^2^ and the Sverdrup mechanism^40^, changes in warm pool sea level also explained how El Niño events affect tropical circulation. On the other hand, Zhang and Clarke^42^ explored observed salinity and temperature patterns in the western equatorial Pacific and discovered the importance of sea level and ocean current responses to freshwater flux during ENSO since the late 1990s.

In summary, El Niño-related anomalies in tropical trade winds and mid-latitude westerlies regulate tropical and subtropical circulation in the North Pacific. The impacts of El Niño on tropical circulation include the strengthening of the North Equatorial Current, Mindanao Current, Indonesian Throughflow, and North Equatorial Countercurrent, while the Kuroshio Current weakens. Although these ocean currents undergo individual changes influenced by various factors, variations in warm pool sea levels provide a relatively simple and universally applicable explanation, which is convenient for evaluating the impact of El Niño events on tropical circulation in climate models. On the other hand, we have provided additional analysis based on current observations of the insufficient understanding of the subtropical Kuroshio. This finding clarifies that the impact of El Niño strengthens the subtropical Kuroshio Current, altering the current community perspective. While variations in the tropical Kuroshio Current can be represented using the NECBL or warm pool sea surface height, the subtropical Kuroshio Current is better described using the wind stress curl resulting from El Niño teleconnections.

## Implications of El Nino diversity

ENSO events varied greatly in amplitude, spatial pattern, and temporal evolution, leading to large differences known as ENSO diversity and complexity. These differences have crucial implications for ENSO teleconnections and societal impacts^34,43^. Certain El Niño events, such as the 1997/1998 Eastern Pacific (EP) El Niño, exhibited notable warming in the eastern Pacific. In contrast, events such as the 2004/2005 Central Pacific (CP) El Niño, displayed stronger central Pacific warming. The different types of El Niño events are known to have varying impacts on regional climates^11,34,44^, and we are intrigued by whether factors such as warm pool sea levels and mid-latitude wind stress curl are applicable in explaining the effects of different types of El Niño events on circulation.

By linearly regressing sea surface currents to the Niño 3 index (Fig. 5a and Extended Data Fig. 7), a proxy of EP El Niño, we found that most Pacific Ocean currents strengthened, including the North Equatorial Current, the Mindanao Current, the North Equatorial Countercurrent, the Indonesian Throughflow, the subtropical Kuroshio Current, and the Kuroshio Extension. On the other hand, the tropical Kuroshio Current weakened (Fig. 5a and Extended Data Fig. 7). Notably, changes in these ocean currents became more extreme when using Niño 4 (Fig. 5b and Extended Data Figs. 7), a proxy of the CP El Niño years. This discovery has critical implications, considering the known increase in the frequency of CP El Niño events due to multidecadal modulation^45^ and/or global warming^26^. On the other hand, global warming also increases the frequency of extreme El Niño and La Niña events^46^. The impact of El Niño events on ocean circulation patterns might become more extreme under a warming scenario.

The differences in the impacts of EP and CP El Niño events on the Northwest Pacific circulation could be attributed to the associated wind stress curl anomalies and warm pool variations. During CP El Niño years, the subtropical wind stress curl became more anomalously negative than that during EP El Niño years because CP El Niño events were more closely associated with the strengthening of midlatitude westerlies (Extended Data Figs. 8 and 9). Compared with EP El Niño years, CP El Niño years exhibited more negative wind stress curl anomalies, leading to a large enhancement of the subtropical Kuroshio Current and Kuroshio Extension (Fig. 5b–d, Extended Data Fig. 7). In the tropics, compared to EP El Niño years, CP El Niño years were more critically associated with pronounced decreases in warm pool sea levels (Fig. 5e,f). This finding provides additional sea level differences across the North Equatorial Current, the Mindanao Current, the North Equatorial Countercurrent, and the Indonesian Throughflow, enhancing their intensities through geostrophic balance. On the other hand, during CP El Niño years, the reduction in sea level differences across the Kuroshio Current in the tropics was more pronounced, leading to a weaker tropical Kuroshio Current compared to EP El Niño years. These results indicated that changes in the warm pool induced by trade winds and variations in the subtropical wind stress curl caused by westerlies could explain different types of El Niño events and their mechanisms of action on tropical and subtropical ocean circulations.

## Discussion and conclusion

Our conclusion is that the impact of El Niño on the Northwest Pacific can be divided into tropical and subtropical dynamic processes. The tropical impact can be explained by changes in warm pool sea levels, while the subtropical impact can be attributed to variations in the wind stress curl. In the tropical North Pacific during El Niño years, enhanced oceanic circulation includes the North Equatorial Current (11 to 20%), Mindanao Current (20 to 25%), North Equatorial Countercurrent (25 to 30%), and Indonesian Throughflow (10 to 15%). On the other hand, the tropical Kuroshio Current weakens (− 5 to −10%). In the subtropical North Pacific, strengthened oceanic circulation includes the subtropical Kuroshio Current (3 to 4%) and Kuroshio Extension (9 to 12%). These mechanisms are applicable to different types of El Niño events, and they are amplified during CP El Niño years compared to EP El Niño years.

In Fig. 6, we examined the impact of ENSO-related wind stress curl anomalies on Rossby waves and the Kuroshio. In the subtropical region, the wind stress curl anomalies (25–30°N, 180°E) intensify the amplitude of Rossby waves (red box in Figure A2b) during El Niño years, reaching the Kuroshio near the Tokara Strait (130°E) with a delay of approximately 3.5 years (Figure A2b). Conversely, in the tropical region, wind stress curl anomalies (145E, 11 N-16 N) during La Niña years intensify the amplitude of Rossby waves (red box in Figure A2c), reaching the Kuroshio near eastern Luzon (127E) with a delay of approximately 6 months (Figure A2c). The speeds of Rossby waves in the subtropics (4.53 cm/s) and tropics (11 cm/s) are consistent with those reported by Chelton et al.^47^. ENSO-excited Rossby waves function in increasing the variability of the Kuroshio.

While researchers have attributed the weakening of the Kuroshio during El Niño to the north‒south migration of the North Equatorial Current bifurcation latitude^2^, our results revealed that the weakening of the Kuroshio occurs only in the tropics, while the subtropical Kuroshio exhibits strengthening attributed to changes in the Pacific wind stress curl. With the intensification of global warming in the future, we anticipate that the impact of El Niño events on ocean circulation in the Northwest Pacific will increase as the frequency of CP-El Niño events will increase^48^. Furthermore, the role of stratification in ocean circulation dynamics is crucial, and the impacts of ocean surface warming, salinity changes, and wind stress alterations on ocean circulation are large. The intensified vertical stratification induced by ocean surface warming, as pointed out by recent studies^49,50^, accelerates upper ocean circulation. We emphasize that the development of more extensive observational systems and climate models is crucial in this regard.

## Methods

Daily sea surface heights (SSH) and surface velocities from AVISO's Ssalto/Duacs, spanning from 1993 to 2022, were processed and distributed in the 'twosat' product by the Copernicus Climate Change Service (C3S, 10.24381/cds.4c328c78). The velocity was calculated based on Absolute Dynamic Topography by AVISO’s Ssalto/Duacs. The Ssalto/Duacs product featured a spatial resolution of 1/4°. These products have delivered high-quality multimission altimetry variables, serving oceanography, climate forecasting, geophysics, and biology communities worldwide.

The sea surface velocity records at a depth of 15 m were provided by the Global Drifter Program (GDP) Surface Velocity Program (SVP) drifters (aoml.noaa.gov), spanning from 1979 to 2022. These buoys were designed to drift with ocean currents, continuously transmitting their position via satellite tracking. They fulfill the requirement for precise and globally distributed in situ observations of mixed layer currents. The SVP drifter data have a temporal resolution of 6-h intervals^52^.

Two long-term ocean reanalysis products were sourced from European Centre for Medium-Range Weather Forecasts (ECMWF)’s Ocean Reanalysis System 4 (ORAS4, https://www.ecmwf.int/en/forecasts/dataset/ocean-reanalysis-system-4) and from National Climate Prediction Center (NCEP)’s Global Ocean Data Assimilation System (GODAS, https://www.cpc.ncep.noaa.gov/products/GODAS/).

Three climate indices were used for regression analysis, including the Ocean Niño index (ONI), Niño 3 index, and Niño 4 index, representing the El Niño phenomena, Eastern Pacific El Niño, and Central Pacific El Niño, respectively. All three indices were downloaded from National Oceanic and Atmospheric Administration (NOAA)/Physical Sciences Laboratory (PSL) (psl.noaa.gov/data/climateindices/list/).

The daily average sea surface temperature (SST) data were sourced from NOAA/PSL's Optimum Interpolation Sea Surface Temperature v2 High Resolution Dataset (OISSTv.2HRD) (https://psl.noaa.gov/data/gridded/data.noaa.oisst.v2.highres.html), covering the period from 1981 to 2022. This SST product offers a spatial resolution of 1/4°. The OISSTv.2HRD integrates data from satellites, ships, buoys, and Argo floats into a global grid. Gaps were filled through optimum interpolation, creating a complete sea surface temperature map. Observations were adjusted using buoy references to account for platform differences and sensor biases^53^. The long-term monthly (SST) data were sourced from NOAA/PSL's Extended Reconstructed SST v5 (ERSST, https://psl.noaa.gov/data/gridded/data.noaa.ersst.v5.html), covering the period from 1854 to 2022. This SST product derived from International Comprehensive Ocean–Atmosphere Data Set (ICOADS) data with missing data were filled in by statistical methods.

The monthly average sea surface winds at 10 m and wind stress data were sourced from the NOAA/PSL's NCEPr1 dataset (https://psl.noaa.gov/data/reanalysis/reanalysis.shtml). Both variables had a spatial resolution of 1.875 degrees. The NCEP/NCAR Reanalysis 1 project utilizes a state-of-the-art analysis/forecast system for data assimilation, incorporating historical data from 1948 to the 2022^54^.

After downloading the drifter velocity data, it was first processed by gridding to achieve a spatial resolution of 0.25 degrees. As we focused on interannual variations, gridded data with less than 12 months in time were ignored. Subsequently, sea surface height, satellite altimeter-derived velocity data, sea surface temperature, and gridded drifter velocity data were processed into monthly averages. Zonal wind stress (u) and merdional wind stress (v) were calculated as the wind stress curl (WSC) based on $\frac{dv}{dx}-\frac{du}{dy}$, and then Sverdrup transport^40^ was obtained through $\frac{1}{\rho \beta }WSC$, and integrated from east to west to obtain the transport of the western boundary currents. $\beta$ is the change in the Coriolis parameter with latitude, and ρ is the density of seawater. Before the data were linearly regressed to the climate indices, anomalies were first identified by removing the annual cycle and then removing the linear trend. The p value and the t-statistic were used to assess statistical significance.

### Supplementary Information

Supplementary Information.

## Data Availability

Data sources are provided within the “Method” section.
