# Supplementary material for: Northwestern Pacific Oceanic circulation shaped by ENSO
Source: Sci Rep. 2024 May 22;14:11684. doi: 10.1038/s41598-024-62361-z (PMC11111802; doi:10.1038/s41598-024-62361-z)
Supplement: Supplementary file 1 — Supplementary Information. [file 41598_2024_62361_MOESM1_ESM.docx]

Extended Data for “Northwestern Pacific oceanic circulation shaped by ENSO”

**Authors:** You-Lin Wang^1*^, Fei-Fei Jin^2^, Chau-Ron Wu^1,3*^, Bo Qiu^4^

*^1^Research Center for Environmental Changes, Academia Sinica, Taipei, Taiwan*

*^2^Department of Atmospheric Sciences, University of Hawaii at Manoa, Hawaii, USA*

*^3^Department of Earth Sciences, National Taiwan Normal University, Taipei, Taiwan*

*^4^Department of Oceanography, University of Hawaii at Manoa, Hawaii, USA*

**
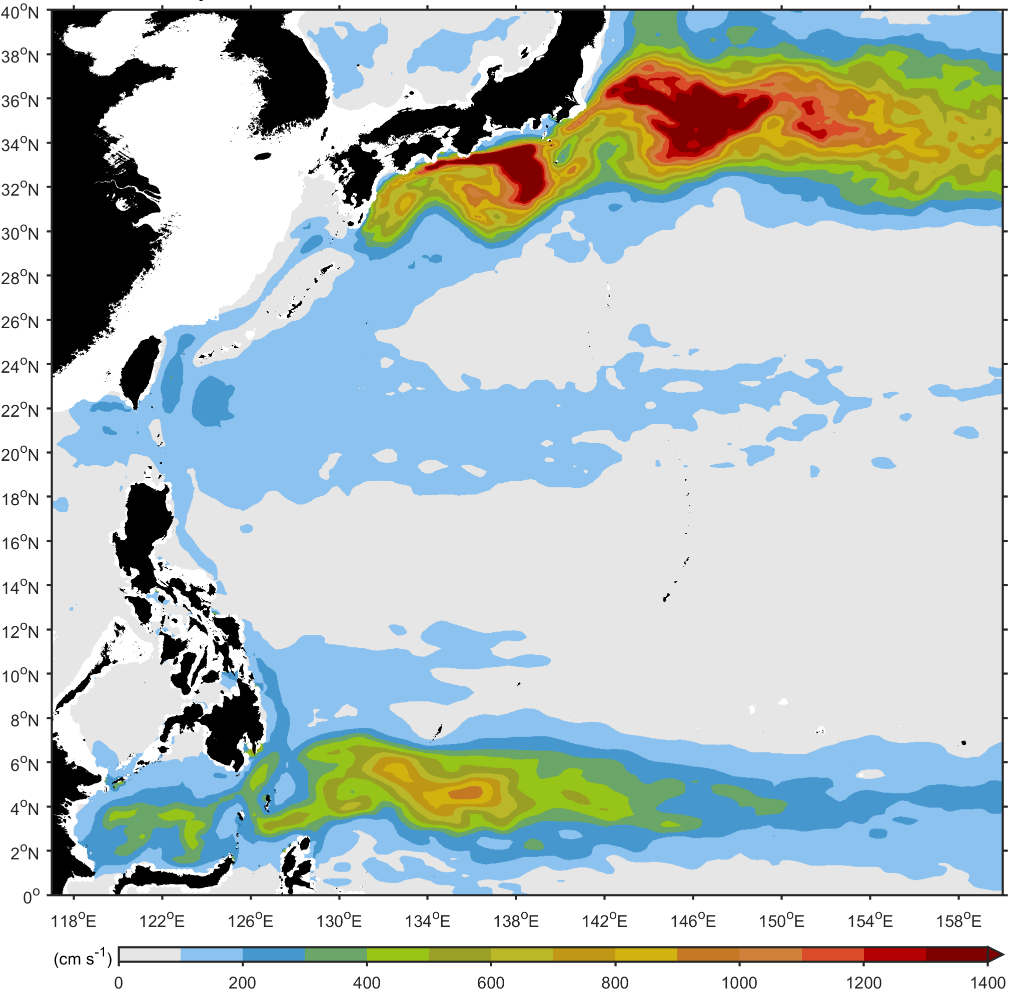
**

**Extended Data Fig. 1.** Variance of 1993–2022 oceanic speed anomaly (based on AVISO).

**
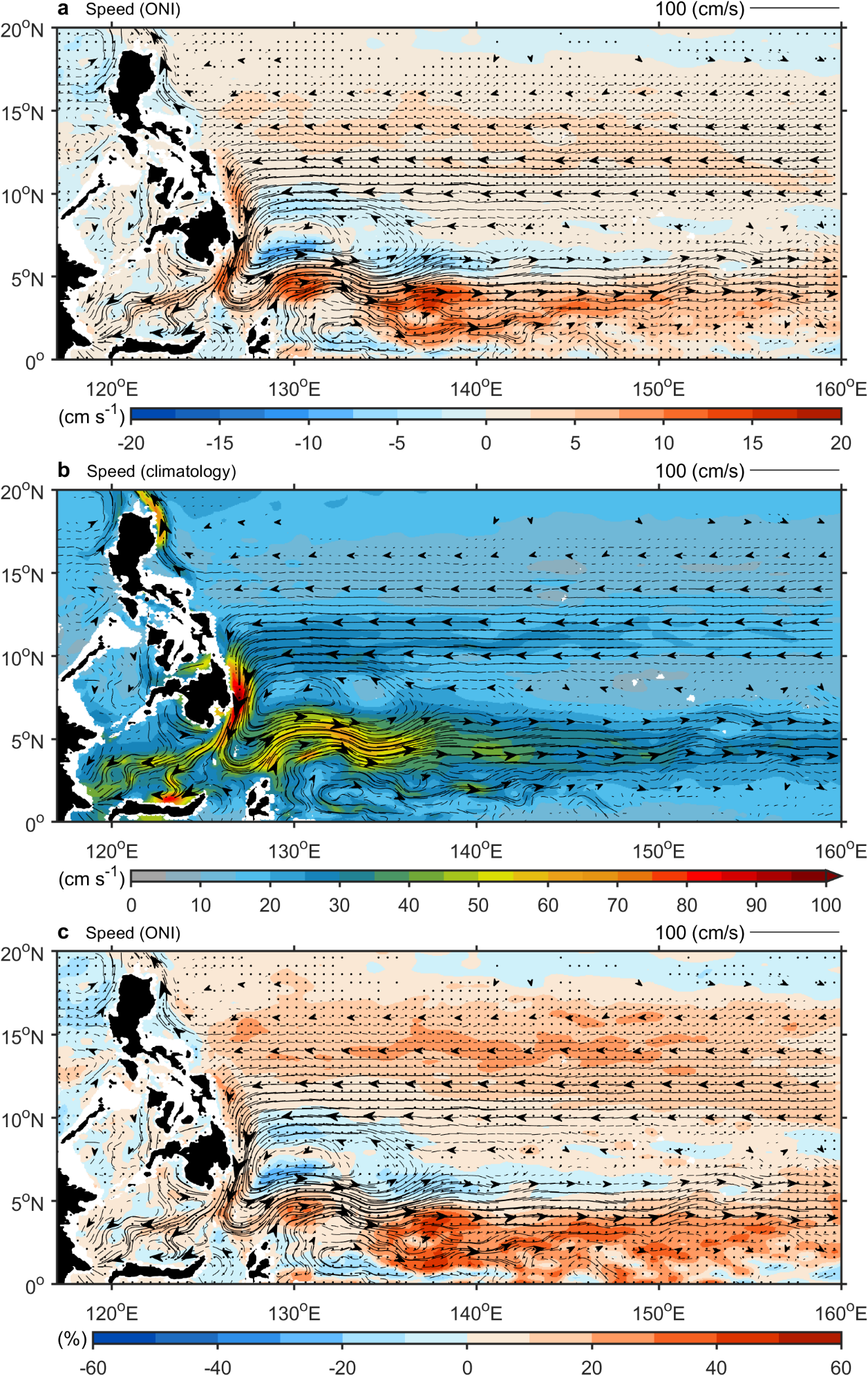
**

**Extended Data Fig. 2. a,** Shading shows 1993–2022 sea surface velocity anomaly regressed onto ONI. Streamlines and arrows depict the mean sea surface circulation. **b,** Same as **a,** but shading shows mean sea surface speed. **c,** Same as **a,** but the shading represents the percentage change in sea surface velocity. It is obtained by dividing the change **a** by the average **b** and multiplying by 100. Black dots signify statistical significance above the 90% confidence level

**
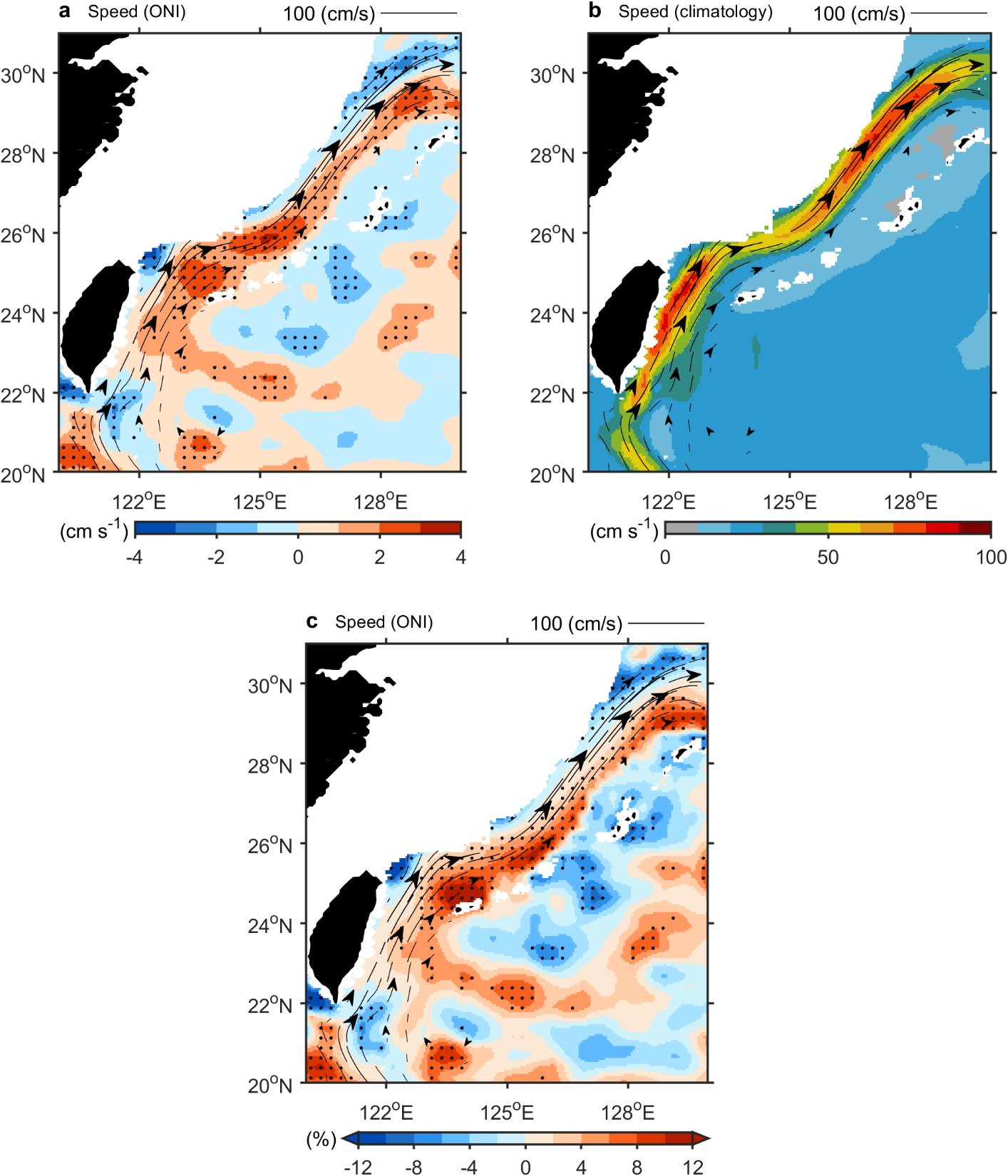
**

**Extended Data Fig. 3.** Same as **Extended Data Fig. 2**, but the area is from the eastern Taiwan to the East China Sea.

**
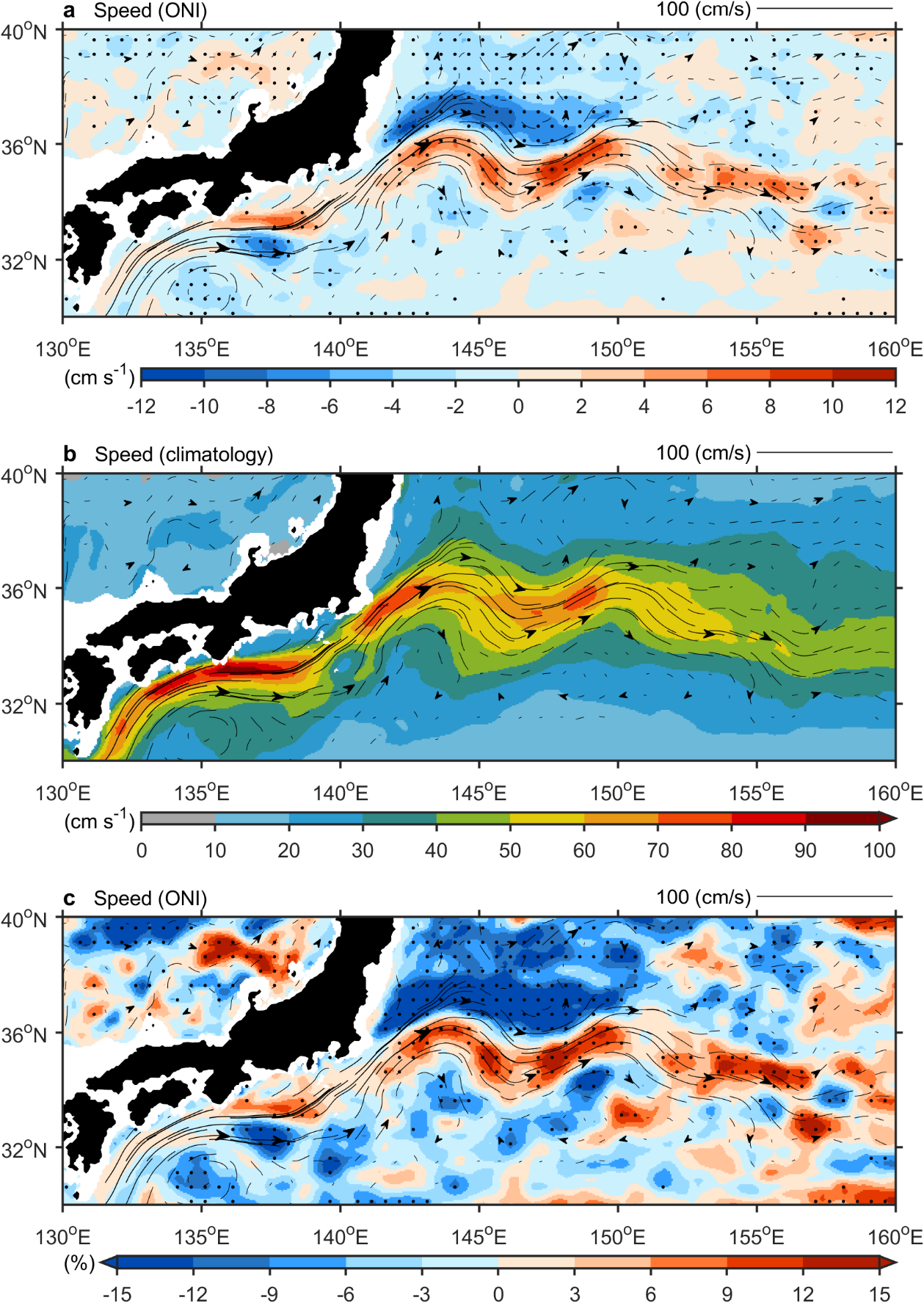
**

**Extended Data Fig. 4.** Same as **Extended Data Fig. 2**, but the area is from the southern Japan to the Kuroshio Extension.

**
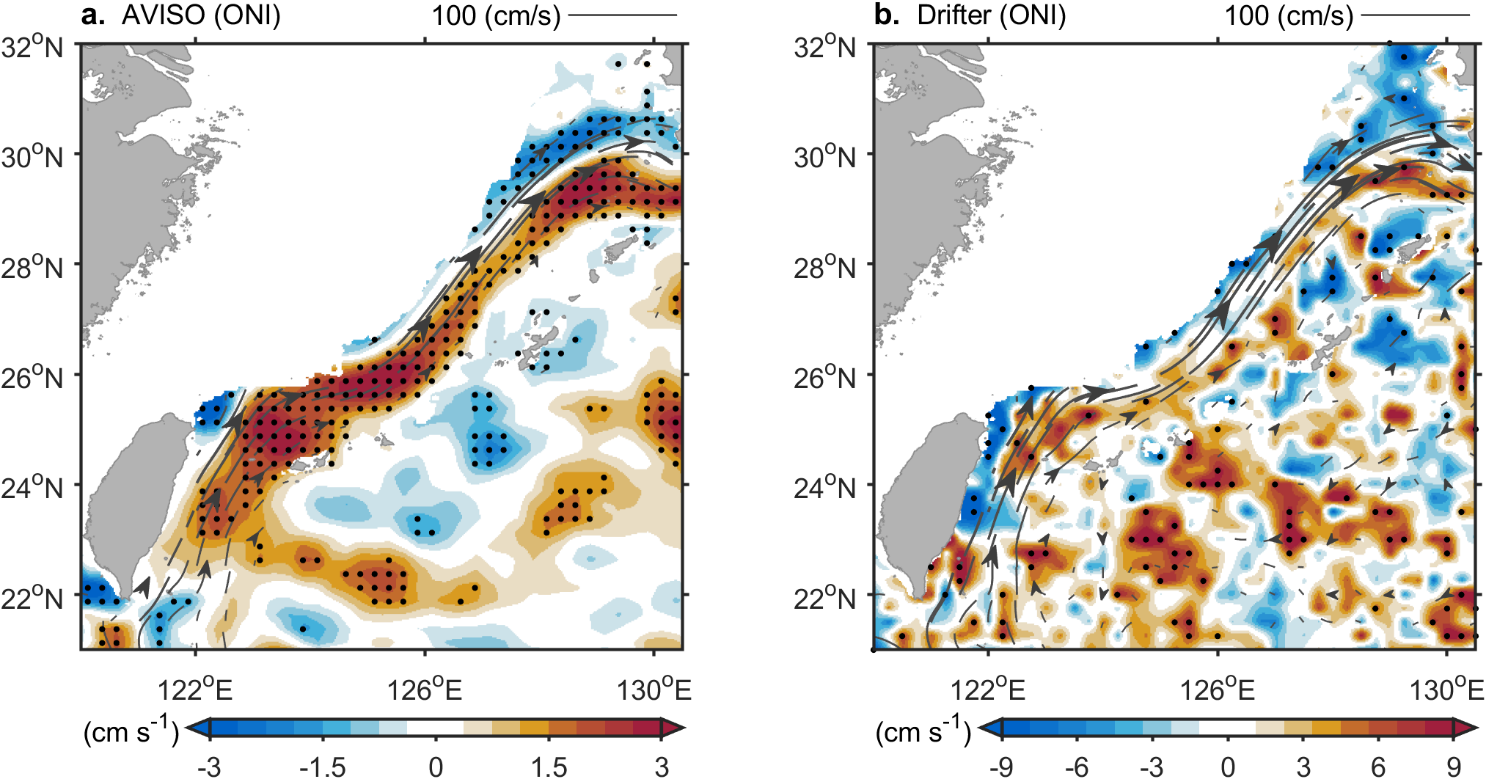
**

**Extended Data Fig. 5.** **a,** The shading shows AVISO's sea surface velocity linear regression onto the ONI index. The black dots indicate statistical significance above the 90% confidence level. The arrows and streamlines represent the average velocity. **b,** Same as **a,** but is velocity of drifter.

**
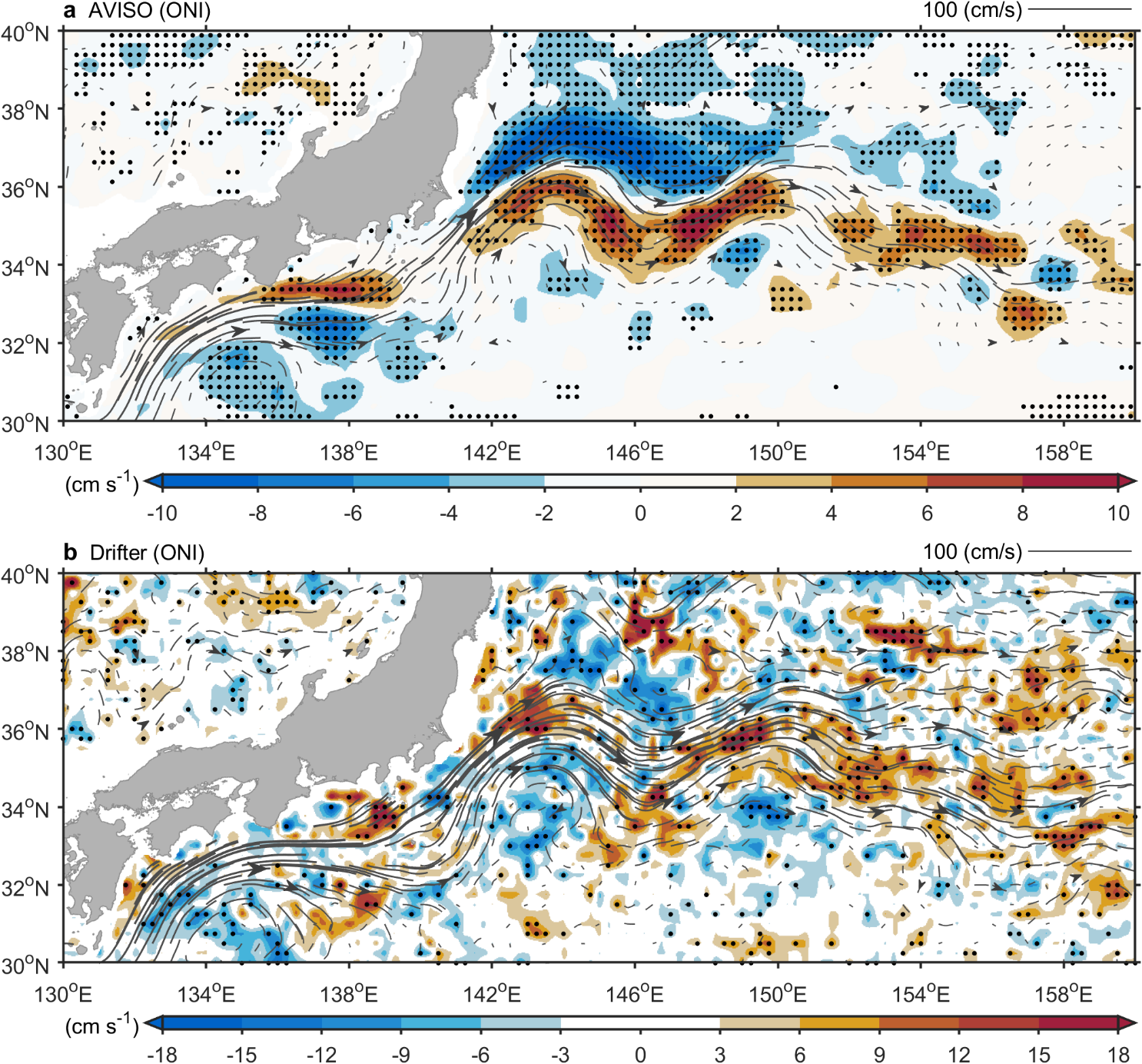
**

**Extended Data Fig. 6.** Same as **Extended Data Fig. 5**, but the area is from the southern Japan to the Kuroshio Extension.

**
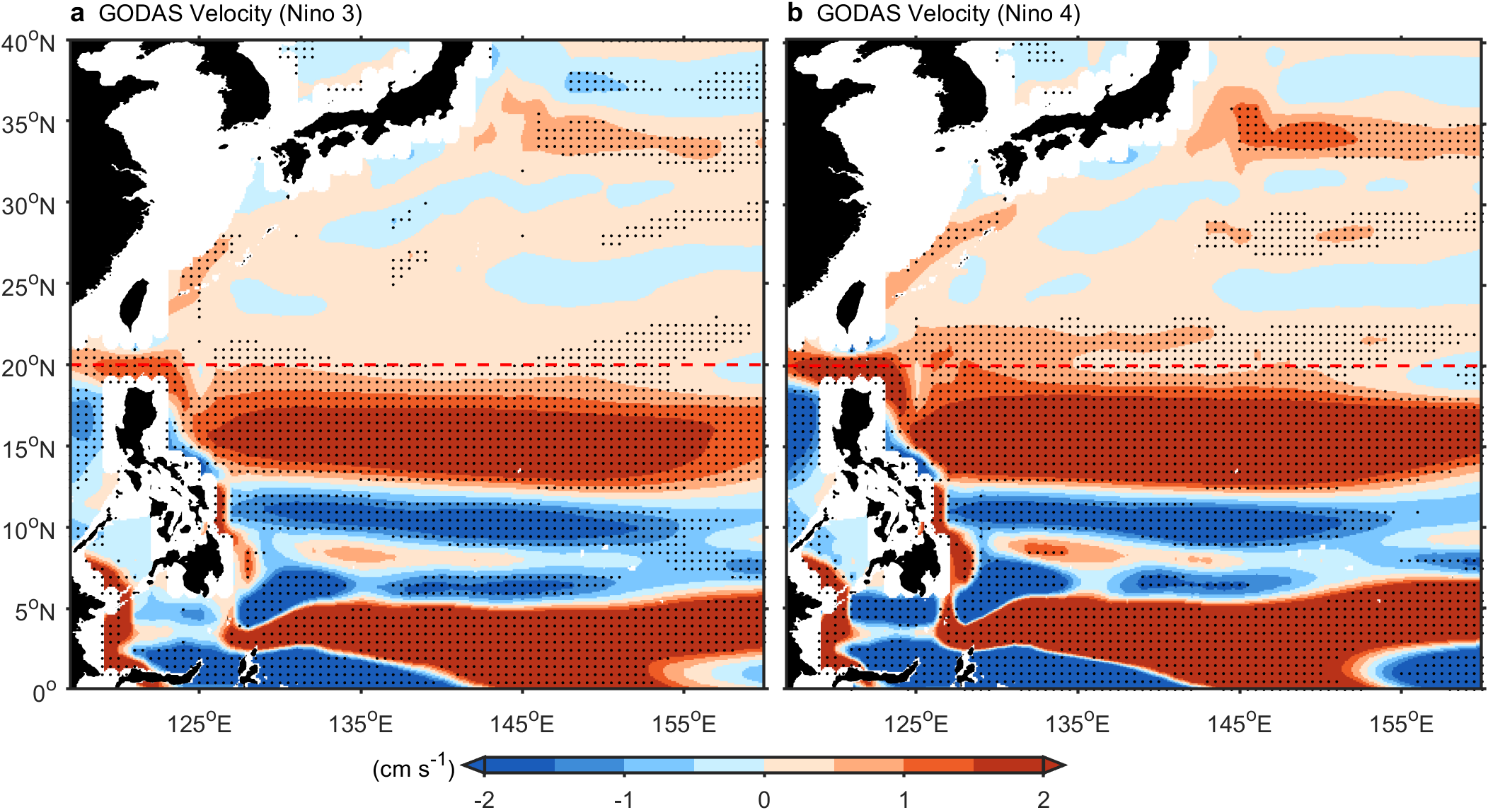
**

**Extended Data Fig. 7.** (a) Shading shows sea surface velocity anomaly (1958 to 2022, upper 200 m) regressed onto the Niño 3 (proxy of EP El Niño). (b) Similar with (a), but using Niño 4 index (proxy of CP El Niño). Black dots signify statistical significance above the 90% confidence level.

**
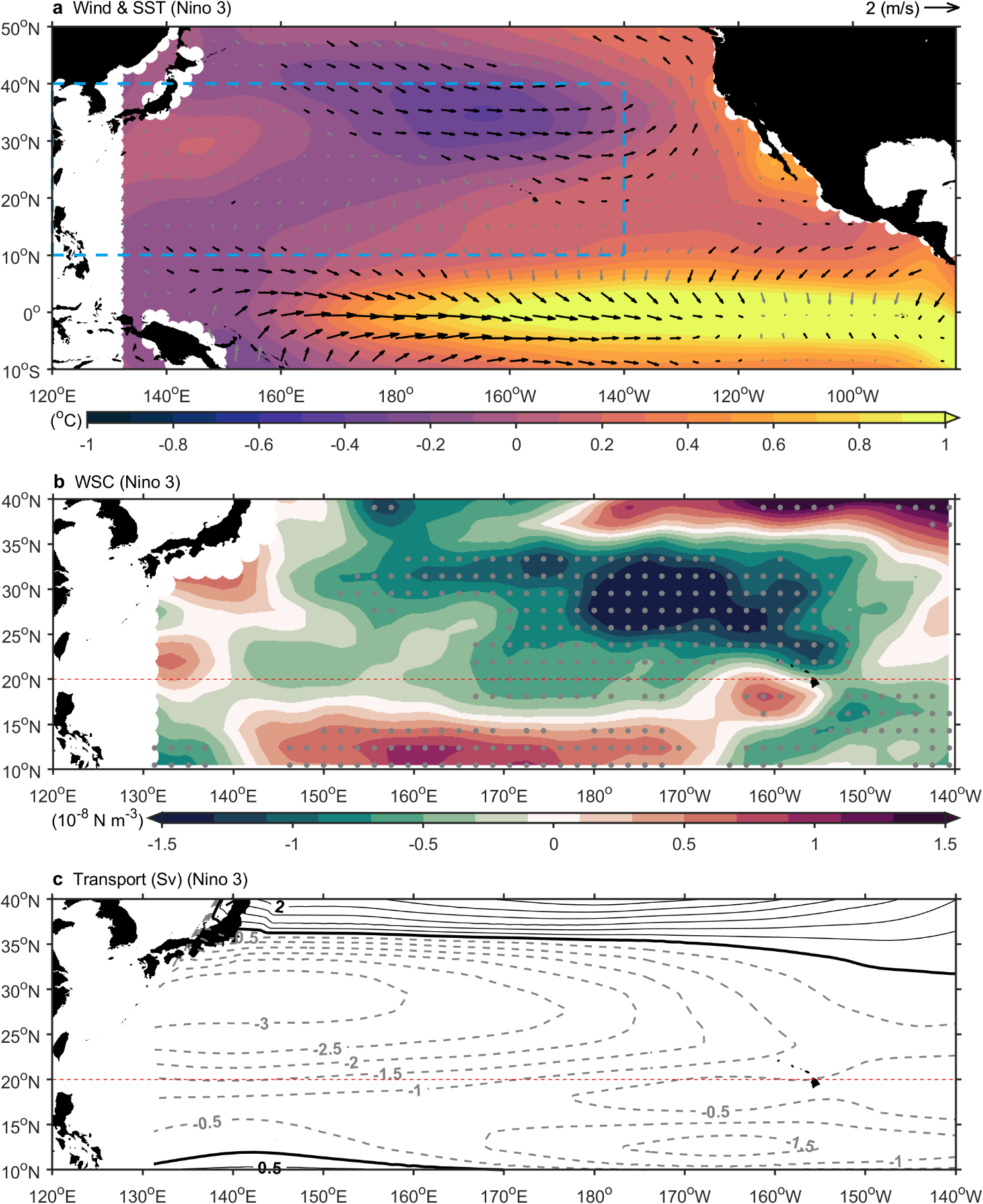
Extended Data Fig. 8. a,** Shading shows 1958–2022 sea surface temperature anomaly regressed onto Niño 3 index. Black and gray arrows indicate significant 10 m winds regression above and below the 99% confidence level. The blue box represents the plotting range of Figs. b and c (120°E–140°W, 10°N–40°N). **b,** Shading represents 1958–2022 wind stress curl anomaly linear regression onto ONI, with gray dots indicating statistical significance above the 99% confidence level. **c,** Contour lines represent linear regression of 1958-2022 North Pacific Sverdrup transport onto the ONI index (contour interval: 0.75 Sv). Negative values indicate southward transport, while positive values represent northward transport. **b**–**c,** Red dash line in both figures marks the boundary between tropical and subtropical regions at 20°N.

**
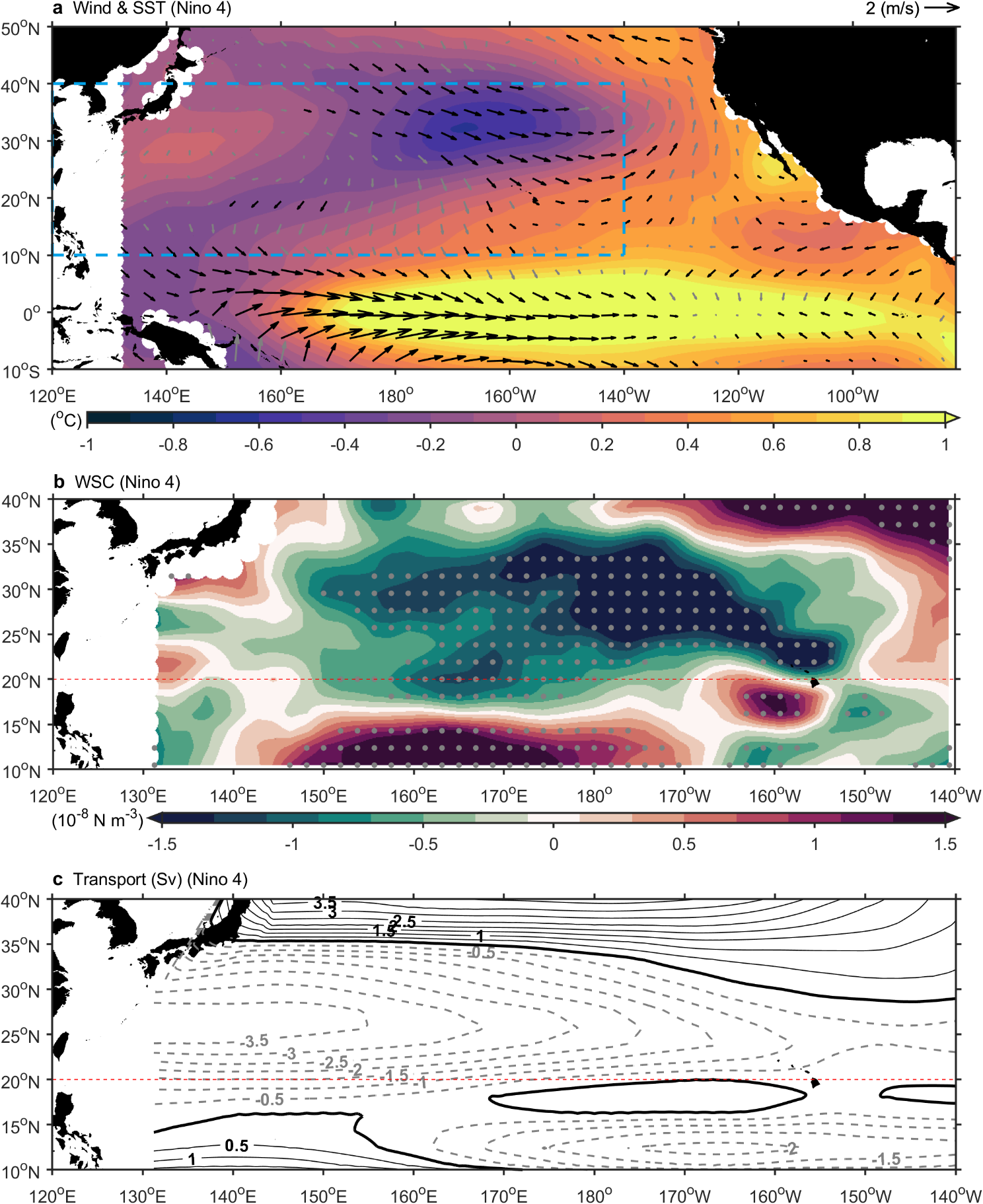
**

**Extended Data Fig. 9.** Same as **Extended Data Fig. 8**, but is regressed onto Niño 4 index.
